# Characterization of Novel MRI Findings in Idiopathic Granulomatous Mastitis: Diagnostic Significance and Clinical Perspectives

**DOI:** 10.1155/tbj/8058328

**Published:** 2026-04-22

**Authors:** Fatih Işık, Mehmet Eren Öztürk, Fatih Alper

**Affiliations:** ^1^ Department of Radiology, Erzurum Regional Training and Research Hospital, Erzurum, Türkiye, erzurumbeah.gov.tr; ^2^ Department of Radiology, Atatürk University Faculty of Medicine, Erzurum, Türkiye, atauni.edu.tr

**Keywords:** breast diseases, granulomatous, magnetic resonance imaging, mastitis

## Abstract

**Objectives:**

Idiopathic granulomatous mastitis (IGM) is an uncommon, benign, chronic inflammatory breast disease that frequently mimics carcinoma both clinically and radiologically, posing diagnostic challenges. While classical imaging manifestations have been described, the full spectrum of MRI features remains incompletely characterized. This study aimed to identify and characterize novel MRI descriptors of histopathologically confirmed IGM and assess their reproducibility and potential clinical implications.

**Methods:**

In this retrospective single‐center study, 45 consecutive women with biopsy‐proven IGM were evaluated using 1.5 T breast MRI. Imaging protocols included T2‐weighted fat‐suppressed, T1‐weighted, diffusion‐weighted, and dynamic contrast‐enhanced sequences. Two experienced radiologists, blinded to clinical and pathological data, independently reviewed all cases for classical and novel MRI features. Interobserver agreement was assessed using raw agreement and Cohen’s *κ* statistics. Associations between imaging descriptors and clinical‐demographic variables were tested with nonparametric methods.

**Results:**

The most common MRI pattern was a heterogeneously enhancing mass with concomitant nonmass enhancement (NME) (75.6%), followed by rim‐enhancing microabscesses (62.2%) and isolated NME (48.9%). Four novel MRI features were identified: spherical transformation of mammary fat lobules (62.2%), disruption of fibroglandular tissue architecture (73.3%), ring‐like enhancement with asymmetric wall morphology (46.6%), and nipple‐areolar complex retraction (11.1%). Interobserver reproducibility was high (*κ* = 0.786–1.000; raw agreement > 90%). Novel descriptors, particularly spherical lobule transformation, fibroglandular disruption, and ring‐like enhancement, were strongly associated with NME (all *p* < 0.001), but not with age or lesion laterality.

**Conclusion:**

These imaging descriptors may contribute to a better radiologic understanding of the morphological manifestations of IGM and may assist radiologists in recognizing imaging patterns associated with granulomatous inflammation. However, given the retrospective design and absence of a comparative cohort, these findings should be considered exploratory and hypothesis‐generating.

## 1. Introduction

Idiopathic granulomatous mastitis (IGM) is an uncommon, benign, chronic inflammatory condition of the breast, primarily impacting women of childbearing age. Initially identified by Kessler and Wolloch in 1972, it is characterized histologically by noncaseating granulomatous inflammation localized to the mammary lobules, with no signs of infection or systemic illness [1]. Although IGM is generally harmless, it often resembles breast cancer in both clinical and radiological assessments, causing diagnostic confusion and occasionally resulting in unnecessary surgical procedures [2–4].

Although histopathological confirmation remains the gold standard for diagnosis, imaging is essential for the early detection, assessment of disease extent, and tracking of treatment progress [5]. Ultrasound often shows irregular hypoechoic lesions with tubular extensions or sinus tracts, while MRI has become crucial for complex cases. MRI findings may include nonmass enhancement (NME), rim‐enhancing microabscesses, skin thickening, and ductal irregularities; however, these features are not definitive and can resemble those seen in malignancies or infectious mastitis [6–9].

Corticosteroids are the cornerstone of therapeutic treatment. Recent studies have shown that local steroid injections are effective and have indicated that their results are similar to those of systemic treatments, with the added benefit of lower systemic toxicity [10]. Consensus guidelines and ultrasonography‐based staging systems have underscored the significance of a multidisciplinary approach and the necessity for precise imaging markers to forecast treatment outcomes [11, 12].

Despite this, the comprehensive range of MRI findings in IGM has not been thoroughly investigated. Discovering new imaging descriptors that hold pathophysiological significance could enhance diagnostic precision, improve the correlation between radiologic and pathologic findings, and aid in making personalized treatment decisions. This research sought to identify novel and less commonly reported MRI characteristics of histopathologically confirmed IGM and assess their potential impact on diagnosis and clinical practice.

## 2. Materials and Methods

### 2.1. Study Design and Patient Selection

This retrospective study took place at a single tertiary referral center from January 2020 to December 2023. January 2020 was selected as the starting point of the study period because standardized breast MRI acquisition protocols and reporting practices were implemented in our institution at that time, ensuring methodological consistency throughout the study period.

The study included 45 consecutive female patients who had histopathologically confirmed IGM. Despite receiving appropriate broad‐spectrum antibiotics, such as beta‐lactams, macrolides, and fluoroquinolones, for a minimum of 10–14 days, all patients continued to experience symptoms. A lack of response was characterized by ongoing pain, redness, swelling, or no reduction in radiological findings on ultrasonography following treatment [4, 13]. Infectious mastitis was ruled out through clinical assessment and laboratory tests, including a complete blood count and inflammatory markers. Tuberculosis was dismissed as a possibility due to negative results from tuberculin skin tests in all patients, while sarcoidosis was excluded based on clinical examination and chest X‐rays. These exclusion procedures aligned with current international consensus guidelines [11]. Only cases involving one side were considered, allowing the unaffected breast on the opposite side to act as an internal control. This approach was intended to minimize interpretive bias when identifying new MRI features. This method was selected to improve the reliability of morphological comparisons between affected and unaffected tissues.

### 2.2. Ethical Approval

This retrospective study was approved by the Institutional Review Board of Atatürk University Faculty of Medicine (Erzurum, Türkiye; Approval No: B.30.2.ATA.0.01.00/552). The requirement for written informed consent was waived due to the retrospective nature of the study and the use of anonymized patient data. All procedures were conducted in accordance with institutional guidelines and the principles of the Declaration of Helsinki.

### 2.3. MRI Acquisition Protocol

All MRI scans were conducted on a 1.5 T system from Philips Healthcare, located in Best, the Netherlands, utilizing a specialized bilateral breast coil. The patients were positioned face down, and premenopausal women were scheduled for scanning between the 7th and 14th days of their menstrual cycle to reduce hormonal influences on breast tissue.

The protocol included the following sequences:

 Axial T2‐weighted fat‐suppressed imaging
 Axial T1‐weighted spin‐echo imaging
 Diffusion‐weighted imaging (DWI) with ADC mapping
 Dynamic contrast‐enhanced (DCE) T1‐weighted 3D gradient‐echo (1 precontrast + 5 postcontrast phases)
 Postcontrast axial and sagittal T1‐weighted fat‐suppressed imaging

A gadolinium‐based contrast agent (0.1 mmol/kg) was administered intravenously at 2 mL/s, followed by a 20 mL saline flush. The total acquisition time was approximately 20–25 min, with the field of view encompassing the axillary and supraclavicular regions.

The choice of MRI sequences was guided by their complementary functions in assessing inflammatory breast conditions. T2‐weighted imaging with fat suppression improves the detection of edema, sinus tracts, and microabscesses, which are frequently observed in IGM. DWI combined with ADC mapping was incorporated to offer a quantitative evaluation of tissue cellularity and diffusion restriction, aiding in distinguishing between granulomatous inflammation, cancerous lesions, and infectious processes [7]. DCE imaging was utilized to evaluate the vascularity of lesions and the kinetics of enhancement, which are crucial for detecting patterns of NME, rim enhancement, and ring‐like enhancement morphology [5, 8, 9]. NME distribution and internal enhancement patterns were classified in accordance with the ACR BI‐RADS MRI lexicon (5th edition) [9]. These sequences collectively enabled a thorough evaluation of both traditional and new imaging characteristics of IGM.

### 2.4. Image Analysis

All MRI datasets were independently reviewed by two breast radiologists with 8 and 20 years of experience, respectively, who were blinded to the clinical and histopathological data. Both radiologists routinely interpret breast MRI examinations at a tertiary referral center and have experience in inflammatory breast diseases, including IGM. The senior radiologist, with 20 years of experience in breast imaging, has substantial clinical experience in IGM and has been involved in ultrasound‐guided intra‐ and perilesional steroid injections for approximately 10 years. During this period, approximately 650 patients with IGM were treated at our institution. Image interpretation was performed independently according to predefined imaging descriptors developed for this study, and no formal consensus training session was conducted prior to the evaluations. The following imaging features were systematically assessed.

• Fat lobule morphology (ellipsoid vs spherical)
• Fibroglandular tissue arrangement (organized radial vs. disrupted)
• Enhancement pattern (mass, nonmass, ring‐like)
• Marginal characteristics of enhancing lesions (smooth vs irregular inner/outer margins)
• Nipple‐areolar complex (NAC) retraction (present/absent)

### 2.5. Statistical Analysis

All analyses were conducted using IBM SPSS Statistics (version 26.0; IBM Corp., Armonk, NY, USA). For continuous variables, descriptive statistics were presented as mean ± standard deviation (SD) and range, while categorical variables were described using frequencies and percentages.

To compare groups based on new MRI findings and clinical‐demographic variables, the Mann–Whitney *U* test was utilized for continuous variables like age, while categorical variables such as lesion side and NME presence were analyzed using the chi‐square or Fisher’s exact test. Spearman’s rank test was employed to evaluate correlations when suitable. A *p* value of less than 0.05 was considered statistically significant.

To evaluate reproducibility, the interobserver agreement between the two radiologists was analyzed for all categorical MRI features. Both the raw agreement (percentage of identical ratings) and Cohen’s *κ* coefficient with 95% confidence intervals (CIs) were calculated. The strength of agreement was interpreted using the Landis and Koch benchmarks (*κ* < 0.20, slight; 0.21–0.40, fair; 0.41–0.60, moderate; 0.61–0.80, substantial; 0.81–1.00, almost perfect) [14]. To determine *κ*, 95% CIs were calculated through percentile bootstrap resampling, conducted over 3000 iterations.

Due to the relatively small sample size and the categorical or nonnormally distributed nature of several variables, nonparametric tests such as the Mann–Whitney *U*, chi‐square, and Fisher’s exact tests were chosen. Spearman’s rank correlation was utilized for ordinal data to avoid assuming linear relationships. These methods were considered suitable to reduce the risk of Type I errors and enhance robustness given the characteristics of the data.

Due to the retrospective nature of the study, an initial sample size calculation was not conducted. However, subsequent analysis suggested that the study had adequate power to detect moderate to large effect sizes for the main associations. Nonetheless, the relatively small sample size might have limited the ability to identify weaker associations, especially in the subgroup analyses.

## 3. Results

This retrospective study assessed the breast MRI results of 45 patients diagnosed with IGM through histopathological confirmation. The evaluation uncovered a wide range of imaging characteristics, encompassing both well‐known features and several findings that had not been previously documented.

The average age of participants in the study was 31.18 years, with a standard deviation of 5.77 years, and ages ranged from 21 to 49 years. Analysis of lesion laterality revealed that 24 patients, accounting for 53.3%, had lesions in the right breast, while 21 patients, or 46.7%, had lesions in the left breast. The study’s inclusion criteria excluded any cases of bilateral disease. Table 1 provides a summary of the patients’ demographic details and MRI results.

**TABLE 1:** Demographic characteristics and MRI findings of patients with idiopathic granulomatous mastitis (*n* = 45).

Variable	*n* (%)
Age (years)	31.18 ± 5.77 (range, 21–49)
Lesion laterality	
Right breast	24 (53.3)
Left breast	21 (46.7)
Common MRI features	
Heterogeneously enhancing mass + NME	34 (75.6)
Small T2‐hyperintense rim‐enhancing lesions (microabscess)	28 (62.2)
NME without associated mass (segmental/regional)	22 (48.9)
Novel MRI findings	
Morphological alteration of mammary fat lobules	28 (62.2)
Disruption of fibroglandular tissue architecture	33 (73.3)

### 3.1. Common MRI Features Consistent With Existing Literature

The most commonly seen imaging pattern was a mass with heterogeneous enhancement, accompanied by NME, observed in 34 patients (75.6%). Small T2‐hyperintense lesions with rim enhancement, indicative of microabscess formation, were found in 28 patients (62.2%). NME without an associated mass was also prevalent, appearing in 22 patients (48.9%), and typically exhibited a segmental or regional distribution.

### 3.2. Novel MRI Findings

Beyond the traditional imaging patterns, researchers have identified four new MRI characteristics that have not been previously reported in relation to IGM. These features were consistently observed only in the affected breast and were not present in the normal tissue of the opposite breast, suggesting a possible link to the pathophysiology of IGM.

#### 3.2.1. Morphological Alterations in Mammary Fat Lobules

In 28 patients (62.2%), mammary fat lobules demonstrated a distinct change from their normal ellipsoid configuration to a more spherical morphology, strictly limited to the affected breast (Figure 1).

FIGURE 1(a) Axial unenhanced T1‐weighted maximum intensity projection (MIP) MR image of the right breast demonstrates spherical transformation of mammary fat lobules (white arrow), representing a novel imaging feature of idiopathic granulomatous mastitis (IGM). The contralateral left breast shows normal ellipsoid fat lobules (black arrow) with preserved radial fibroglandular architecture. (b) Corresponding schematic diagram illustrating the spherical configuration of mammary fat lobules and disruption of fibroglandular architecture in the affected breast compared with the normal ellipsoid lobules and preserved glandular organization in the contralateral breast.

**Figure:**
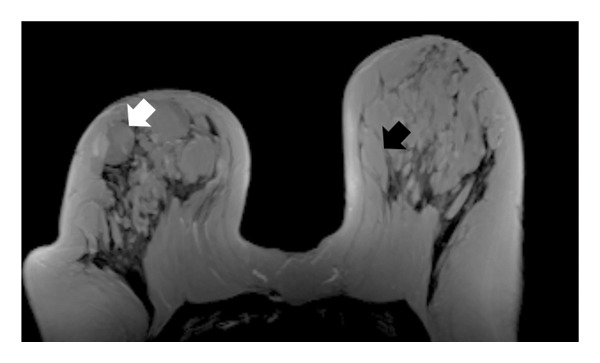
(a)

**Figure:**
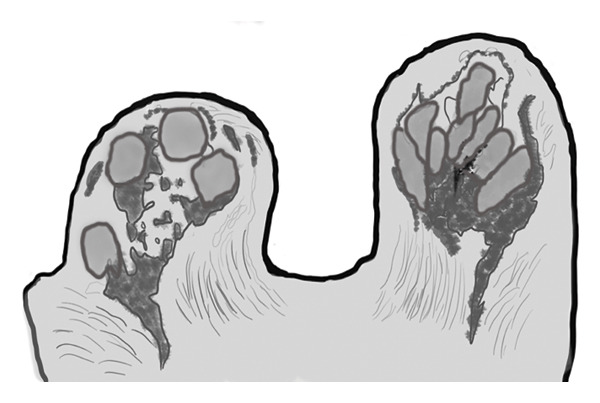
(b)

#### 3.2.2. Disruption of the Fibroglandular Tissue Architecture

In 33 patients (73.3%), the normal radial alignment of the fibroglandular tissue extending toward the NAC was replaced by a dysmorphic and irregular arrangement deviating from the typical spoke‐wheel pattern (Figure 2).

FIGURE 2(a) Axial unenhanced T1‐weighted MR image of the right breast demonstrates disruption of the normal radial alignment of fibroglandular tissue (black arrows). Instead of the typical organized spoke‐wheel pattern extending toward the nipple‐areolar complex, the fibroglandular structures appear irregular and fragmented. The contralateral left breast shows preserved fibroglandular architecture (white arrows). (b) Corresponding schematic diagram illustrating architectural distortion of the fibroglandular tissue in the affected breast compared with the preserved radial organization in the contralateral breast, representing one of the novel MRI features of idiopathic granulomatous mastitis.

**Figure:**
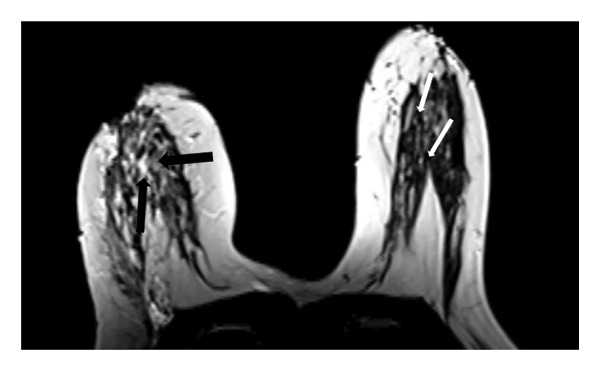
(a)

**Figure:**
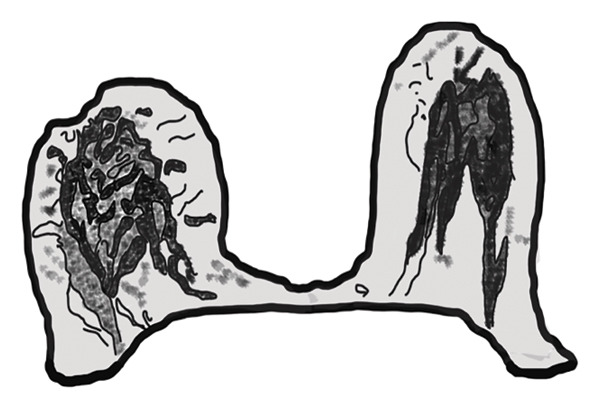
(b)

#### 3.2.3. Ring‐Like Enhancement With Asymmetric Wall Morphologies

In 21 patients (46.6%), postcontrast images revealed ring‐like enhancement characterized by a smooth inner margin and irregular, indistinct outer contour. This enhancement diverges from the classical appearance of uncomplicated abscesses or benign cystic lesions and may reflect granulomatous inflammation with peripheral infiltration (Figure 3).

FIGURE 3(a) Axial contrast‐enhanced T1‐weighted MR image of the right breast demonstrates a ring‐like enhancing lesion characterized by a smooth, well‐defined inner margin (black arrow) and an irregular, indistinct outer margin (white arrow). This asymmetric morphology diverges from the classical appearance of abscesses or cysts and suggests peripheral granulomatous infiltration in IGM. (b) Corresponding schematic diagram illustrating the enhancement pattern, highlighting the contrast between the smooth inner margin and the irregular outer contour.

**Figure:**
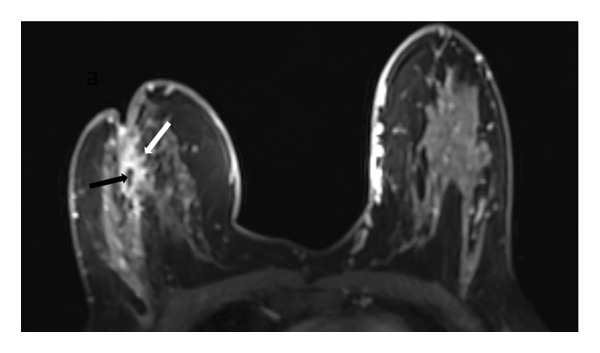
(a)

**Figure:**
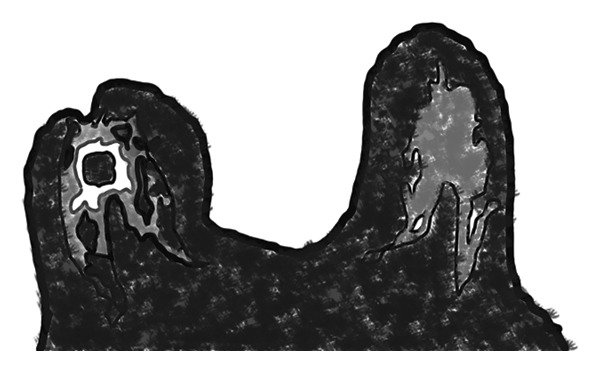
(b)

#### 3.2.4. Retraction of the NAC

NAC retraction was identified in five patients (11.1%). Although described in prior reports as a nonspecific sign, its repeated occurrence in histopathologically confirmed IGM cases in our cohort suggests a more meaningful diagnostic association (Figure 4).

FIGURE 4(a) Axial unenhanced T1‐weighted MR image demonstrates retraction of the nipple‐areolar complex (NAC) in the left breast (arrow), associated with underlying parenchymal architectural distortion. This finding may reflect a reactive fibrotic process in idiopathic granulomatous mastitis. (b) Corresponding schematic diagram illustrating asymmetry of the NAC, with retraction on the affected side compared with the preserved contour on the contralateral breast, along with associated fibroglandular architectural disruption.

**Figure:**
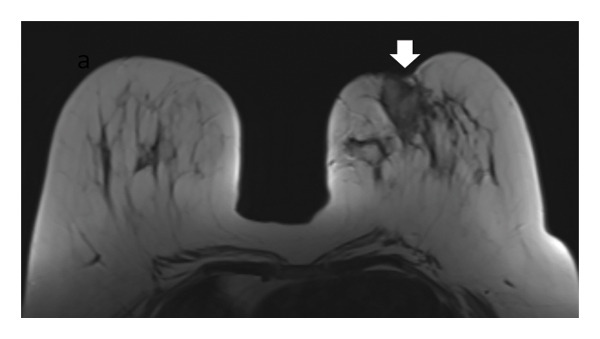
(a)

**Figure:**
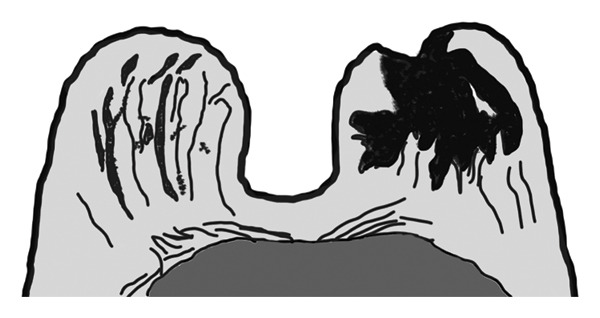
(b)

### 3.3. Interobserver Agreement

Evaluation of interobserver reliability revealed a high level of agreement for all categorical MRI features, ranging from substantial to perfect. The raw agreement percentages varied from 91.1% (95% CI, 78.8%–97.5%) for NME, spherical fat lobule morphology, and fibroglandular disruption to a full 100% (95% CI, 92.1%–100%) for NAC retraction and ring‐like enhancement assessments. Cohen’s *κ* values supported these results, showing substantial agreement for NME (*κ* = 0.786, 95% CI, 0.549–0.952) and fibroglandular disruption (*κ* = 0.795, 95% CI, 0.579–0.951), almost perfect agreement for spherical fat lobule morphology (*κ* = 0.819, 95% CI, 0.634–0.955), and perfect agreement for both NAC retraction (*κ* = 1.000) and ring‐like enhancement (*κ* = 1.000) (Table 2).

**TABLE 2:** Interobserver agreement between two radiologists for categorical MRI features of idiopathic granulomatous mastitis (*n* = 45).

Feature	Agreement (*n*/*N*)	Agreement %	95% CI (Clopper–Pearson)	Cohen’s *κ*	95% CI (bootstrap)
NME	41/45	91.1%	78.8%–97.5%	0.786	[0.549–0.952]
NAC retraction	45/45	100%	92.1%–100%	1.000	—
Spherical fat lobule	41/45	91.1%	78.8%–97.5%	0.819	[0.634–0.955]
Disruption of fibroglandular tissue	41/45	91.1%	78.8%–97.5%	0.795	[0.579–0.951]
Ring‐like enhancement	45/45	100%	92.1%–100%	1.000	[1.000–1.000]

### 3.4. Associations With Clinical and Imaging Parameters

Further analyses were conducted to assess the relationships between new MRI characteristics and clinical‐demographic factors. There were no notable differences in age between patients with and without spherical fat lobule transformation (*p* = 0.944), fibroglandular disruption (*p* = 0.503), ring‐like enhancement (*p* = 0.673), or NAC retraction (*p* = 0.703). Likewise, the side of the lesion (right versus left breast) did not show a significant correlation with any of the new features (all *p* > 0.16).

Conversely, NME exhibited a strong and significant association with the transformation of spherical fat lobules, disruption of fibroglandular tissue, and ring‐like enhancement (all *p* < 0.001), but it was not linked to NAC retraction (*p* = 1.000). These findings indicate that although most new descriptors are independent of demographic factors, some are closely related to established enhancement patterns, highlighting their potential pathophysiological significance in IGM (Table 3).

**TABLE 3:** Associations between novel MRI features and clinical‐demographic variables in patients with idiopathic granulomatous mastitis (*n* = 45).

Comparison	Test	*p* value
Age vs. spherical fat lobule	Mann–Whitney *U*	0.944
Age vs. fibroglandular disruption	Mann–Whitney *U*	0.503
Age vs. ring‐like enhancement	Mann–Whitney *U*	0.673
Age vs. NAC retraction	Mann–Whitney *U*	0.703
Lesion side vs. spherical fat lobule	Chi‐square/Fisher	0.356
Lesion side vs. fibroglandular disruption	Chi‐square/Fisher	0.329
Lesion side vs. ring‐like enhancement	Chi‐square/Fisher	0.373
Lesion side vs. NAC retraction	Chi‐square/Fisher	0.169
NME vs. spherical fat lobule	Chi‐square/Fisher	*p* < 0.001
NME vs. fibroglandular disruption	Chi‐square/Fisher	*p* < 0.001
NME vs. ring‐like enhancement	Chi‐square/Fisher	*p* < 0.001
NME vs. NAC retraction	Chi‐square/Fisher	1.000

## 4. Discussion

In line with previously documented imaging features, the most common MRI pattern observed in our group was a mass with heterogeneous enhancement accompanied by NME. This observation is consistent with earlier studies by Sripathi et al. and Kocaoglu et al., who highlighted the frequent occurrence of both mass‐like and NME in IGM [4, 15]. In 28 patients, small lesions that appeared hyperintense on T2‐weighted images and showed rim enhancement, suggesting microabscesses, were detected. Such findings are commonly recognized as typical yet nonspecific signs of localized inflammation in IGM [4, 5, 13, 15]. Moreover, isolated NME without an accompanying mass was identified in 22 patients, typically appearing in a segmental or regional pattern. This aligns with the findings of Zhao et al. and Soylu Boy et al., who noted that isolated NME can resemble ductal carcinoma in situ or invasive carcinoma [7, 8]. Interestingly, the percentage of patients with isolated NME in our study was somewhat higher than that reported in previous MRI research, which may indicate variations in case selection or the stage of the disease at the time of imaging.

In addition to the well‐known characteristics, our research introduced several new MRI descriptors that have not been previously identified in IGM. Notably, the spherical transformation of mammary fat lobules was recognized as a unique feature of the condition. This change likely results from the combined impact of granulomatous inflammation, surrounding edema, and reactive fibrosis, which together cause isotropic compression of the lobular units. From a histopathological perspective, lobulocentric granulomatous infiltration, multinucleated giant cells, and perilesional fibrosis offer a plausible explanation for this morphological alteration [3, 16–18]. In a similar manner, the alteration of the typical radial arrangement of fibroglandular tissue might be linked to histological signs of glandular damage and fibrotic changes, a phenomenon noted in other chronic inflammatory breast disorders [17, 19, 20]. A distinctive characteristic, the ring‐like enhancement pattern featuring a smooth inner edge and an uneven outer boundary, might indicate zonal inflammatory reactions with central necrosis and peripheral granulomatous infiltration, differing from typical abscesses or malignancies [5, 17, 21, 22]. In our study group, we frequently noted NAC retraction, which, despite being traditionally seen as nonspecific, appeared without any malignant characteristics, highlighting the importance of interpreting it carefully within the appropriate context [23].

This study has identified new MRI characteristics that seem to have credible correlations with imaging and pathology. For instance, the transformation of spherical lobules indicates perilesional edema and granulomatous infiltration, which disrupts the alignment of adipocytes, while the disruption of fibroglandular tissue reflects lobular distortion and fibrotic remodeling [3, 17]. The appearance of a ring‐like enhancement is indicative of central necrosis surrounded by granulomatous inflammation, aligning with zonal histopathological alterations [5, 21, 22]. The retraction of the NAC might be attributed to fibrosis and scarring in the subareolar stroma, occurring without any malignant infiltration [17]. Additionally, the correlation with ultrasonography supports the biological plausibility of these descriptors: irregular hypoechoic lesions with sinus tracts might be the sonographic equivalent of fibroglandular disruption, while microabscesses that appear as rim‐enhancing T2‐hyperintense areas on MRI align with hypoechoic cavities containing internal debris on ultrasound [24, 25].

In our study, both traditional and new MRI characteristics demonstrated high reproducibility, with interobserver *κ* values ranging from substantial to perfect (0.786–1.000). The raw agreement surpassed 90% for all descriptors assessed, highlighting their robustness. Previous research indicates that *κ* values above 0.75 signify excellent agreement for imaging biomarkers [14]. The notably high level of agreement for spherical lobule transformation, fibroglandular disruption, and ring‐like enhancement underscores their potential as dependable diagnostic indicators of IGM. The complete concordance between NAC retraction and ring‐like enhancement is particularly significant, given their traditional link to malignancy. This suggests that, in the right clinical setting, these observations might be reinterpreted as consistent signs of granulomatous inflammation rather than cancer [5]. The high level of interobserver agreement observed in this study also suggests that these imaging features can be recognized reliably once their morphological characteristics are clearly defined. Nevertheless, further studies including readers with different levels of experience are needed to better determine the learning curve and generalizability of these findings. It should also be emphasized that the interobserver agreement reported in this study was obtained in a highly specialized setting. Both readers were experienced breast radiologists working at a tertiary referral center with substantial institutional experience in the imaging and management of IGM. Consequently, the high reproducibility observed in this cohort may partly reflect this level of expertise and familiarity with the disease. The generalizability of these imaging descriptors and their reproducibility among radiologists with varying levels of experience remain uncertain and should be evaluated in future multicenter studies.

Although histopathological confirmation remains essential for the diagnosis of IGM, imaging plays an important role in the evaluation of complex breast lesions and in differentiating inflammatory conditions from malignancy. Breast MRI is often requested in clinically challenging situations, particularly when ultrasound findings are equivocal or when inflammatory carcinoma cannot be confidently excluded. In such contexts, recognition of characteristic MRI patterns associated with granulomatous inflammation may increase diagnostic confidence and assist radiologists in considering IGM in the differential diagnosis. Consequently, these imaging descriptors may help reduce misinterpretation of inflammatory changes as malignancy and contribute to a more cautious and accurate radiologic assessment.

In addition to their descriptive value, these MRI features may provide further insight into the morphological manifestations of granulomatous inflammation within breast tissue. Recognition of patterns such as spherical fat lobule transformation, fibroglandular disruption, and asymmetric ring‐like enhancement may help radiologists better characterize inflammatory breast lesions during MRI interpretation. However, given the absence of a comparative control group in the present study, these findings should not be interpreted as specific diagnostic markers for differentiating IGM from malignancy or other inflammatory breast diseases. Rather, they should be regarded as preliminary imaging observations that warrant further investigation in prospective studies including appropriate comparison cohorts.

In accordance with these observations, statistical analyses further validate the clinical importance of the newly identified characteristics. The transformation of spherical lobules, disruption of fibroglandular tissue, and ring‐like enhancement showed a strong correlation with NME (all *p* < 0.001), aligning with the hypothesis that they are morphological outcomes of the same inflammatory process. On the other hand, no links were found with the patient’s age or the side of the lesion, suggesting that these features are inherent to the disease’s pathophysiology rather than influenced by demographic factors. Notably, NAC retraction did not show a correlation with NME, implying that it might result from secondary fibrotic remodeling instead of active inflammation [17].

From a differential diagnosis standpoint, these characteristics can aid in differentiating IGM from other similar conditions. While fat necrosis can also alter lobules, it typically results in oil cysts or calcifications instead of round lobules [26, 27]. Inflammatory carcinoma is often characterized by skin thickening and swelling, and it usually progresses rapidly with the presence of axillary lymph node enlargement [28]. Breast abscesses often exhibit peripheral rim enhancement and can have smooth, well‐defined margins. Nonetheless, they may also present with irregular, thickened rims, indicating that rim morphology alone is not a definitive characteristic [29–31]. Therefore, the combination of spherical lobule transformation, disruption of fibroglandular tissue, and asymmetric ring‐like enhancement may represent an imaging pattern associated with granulomatous inflammation. Nevertheless, these observations should be interpreted cautiously and always in conjunction with clinical findings and histopathological confirmation. The combined presence of spherical fat lobule transformation, disruption of fibroglandular architecture, and asymmetric ring‐like enhancement may therefore represent an imaging pattern that favors granulomatous inflammation rather than malignancy. Nevertheless, these findings should always be interpreted in conjunction with clinical evaluation and histopathological confirmation.

In clinical practice, these MRI features may represent morphological manifestations of the underlying inflammatory process and may provide additional context when interpreting complex breast MRI examinations. However, their potential relationship with disease activity or treatment response remains uncertain and requires investigation in future prospective studies [12]. In addition, exploring possible relationships between these imaging findings and the clinical stage of the disease (such as acute inflammatory exacerbation versus chronic phases) may provide further insight into the biological behavior of IGM. Although our results are encouraging, they need to be validated in larger prospective cohorts to ensure their applicability in routine clinical practice. Such studies may help establish MRI‐based markers as useful adjuncts for monitoring disease activity and supporting more individualized and conservative management strategies in patients with IGM.

## 5. Limitations

This research faced several limitations. Firstly, its retrospective and single‐center nature inherently restricts its applicability to broader contexts. Secondly, the study involved a relatively small sample size and lacked a control group of patients with other benign or malignant breast conditions, which prevents drawing definitive conclusions about the specificity of new MRI features. The absence of a control group including patients with inflammatory breast carcinoma, breast abscess, or fat necrosis limits the ability to directly assess the specificity of the proposed MRI descriptors. Future prospective studies including comparative cohorts will be necessary to determine the diagnostic performance of these findings in differentiating IGM from other breast pathologies. Thirdly, the analysis was limited to unilateral cases. While this method allowed the contralateral breast to act as an internal reference, enhancing the identification of new imaging features, it also limited the generalizability of our results. Although bilateral IGM is less frequently encountered in clinical settings, it represents a significant subset of patients, and it is uncertain if the newly identified descriptors would show similar diagnostic consistency in this group. Future prospective studies that include bilateral cases are necessary to verify if these imaging patterns apply across the entire clinical spectrum of IGM. Furthermore, since no formal sample size calculation was performed before data collection, the study might have lacked the power to detect subtle associations. All MRI examinations were performed on a single 1.5 T platform, which may limit generalizability to other field strengths and vendors. This issue should be addressed in future prospective trials with larger, predefined cohort sizes.

## 6. Conclusion

In conclusion, this study identifies four previously undescribed MRI features observed in histopathologically confirmed IGM and demonstrates that these descriptors can be recognized with high interobserver agreement in a specialized setting. While these findings contribute to the radiologic characterization of IGM, the retrospective design and absence of a comparative cohort limit conclusions regarding diagnostic specificity. Therefore, these observations should be considered exploratory and hypothesis‐generating, and further validation in larger prospective multicenter studies including appropriate control populations is necessary.

## Author Contributions

Fatih Işık and Fatih Alper analyzed the ultrasonographic images. Fatih Işık made schematic drawings of the sonographic images. Mehmet Eren Öztürk performed clinical research and participated in literature analysis for the Discussion section. Fatih Işık wrote the manuscript.

## Funding

No funds were used for this study.

## Disclosure

All authors read and approved the final manuscript.

## Ethics Statement

This study was approved by the Institutional Review Board of the Faculty of Medicine, Atatürk University (Erzurum, Türkiye; Approval No. B.30.2.ATA.0.01.00/552). The requirement for written informed consent was waived by the committee owing to the retrospective design and the use of anonymized data, in accordance with institutional policy, national regulations, and the Declaration of Helsinki (1964 and subsequent revisions).

## Consent

Please see the Ethics Statement.

## Conflicts of Interest

The authors declare no conflicts of interest.

## Data Availability

The data that support the findings of this study are available from the corresponding author upon reasonable request.
